# Prenatal diagnosis of Norrie disease after whole exome sequencing of an affected proband during an ongoing pregnancy: a case report

**DOI:** 10.1186/s12881-020-01093-z

**Published:** 2020-10-22

**Authors:** Andrey V. Marakhonov, Irina A. Mishina, Vitaly V. Kadyshev, Svetlana A. Repina, Maria F. Shurygina, Olga A. Shchagina, Natalya N. Vasserman, Tatyana A. Vasilyeva, Sergey I. Kutsev, Rena A. Zinchenko

**Affiliations:** 1 Laboratory of Genetic Epidemiology, Research Centre for Medical Genetics, Moscow, Russian Federation; 2 S. Fyodorov Eye Microsurgery Federal State Institution, Moscow, Russian Federation; 3 N.A. Semashko National Research Institute of Public Health, Moscow, Russian Federation

**Keywords:** Hereditary eye pathology, Clinical heterogeneity, NGS, *NDP*, Norrie disease

## Abstract

**Background:**

Hereditary ophthalmic pathology is a genetically heterogeneous group of diseases that occur either as an isolated eye disorder or as a symptom of hereditary syndromes (chromosomal or monogenic). Thus, a diagnostic search in some cases of ophthalmic pathology can be time- and cost-consuming. The most challenging situation can arise when prenatal diagnosis is needed during an ongoing pregnancy.

**Case presentation:**

A family was referred to the Research Centre for Medical Genetics (RCMG) for childbirth risk prognosis at 7–8 week of gestation because a previous child, a six-year-old boy, has congenital aniridia, glaucoma, retinal detachment, severe psychomotor delay, and lack of speech and has had several ophthalmic surgeries. The affected child had been previously tested for *PAX6* mutations and 11p13 copy number variations, which revealed no changes. Considering the lack of pathogenic changes and precise diagnosis for the affected boy, NGS sequencing of clinically relevant genes was performed for the ongoing pregnancy; it revealed a novel hemizygous substitution NM_000266.3(*NDP*):c.385G > T, p.(Glu129*), in the *NDP* gene, which is associated with Norrie disease (OMIM #310600). Subsequent Sanger validation of the affected boy and his mother confirmed the identified substitution inherited in X-linked recessive mode. Amniotic fluid testing revealed the fetus was hemizygous for the variant and lead to the decision of the family to interrupt the pregnancy. Complications which developed during the termination of pregnancy required hysterectomy due to medical necessity.

**Conclusions:**

Clinical polymorphism of hereditary ophthalmic pathology can severely complicate establishment of an exact diagnosis and make it time- and cost-consuming. NGS appears to be the method-of-choice in complicated cases, and this could substantially hasten the establishment of a diagnosis and genetic risk estimation.

## Background

Hereditary eye pathology is a genetically heterogeneous group of diseases that occur either as an isolated ocular disorder or as a symptom of syndromes (both chromosomal and monogenic). We present here the results of DNA diagnosis in a patient with complex ocular phenotype associated with severe systemic involvement using next-generation sequencing (NGS), not only to perform differential diagnosis but also to determine if such a complex clinical picture has a single etiology or is a result of an interaction of factors that could have both hereditary and environmental origin. The case is much more complicated because the proband’s mother had an ongoing pregnancy, thus the DNA diagnosis was performed with urgent need for genetic risk estimation as well as subsequent prenatal diagnosis.

## Case presentation

**Fig. 1:**
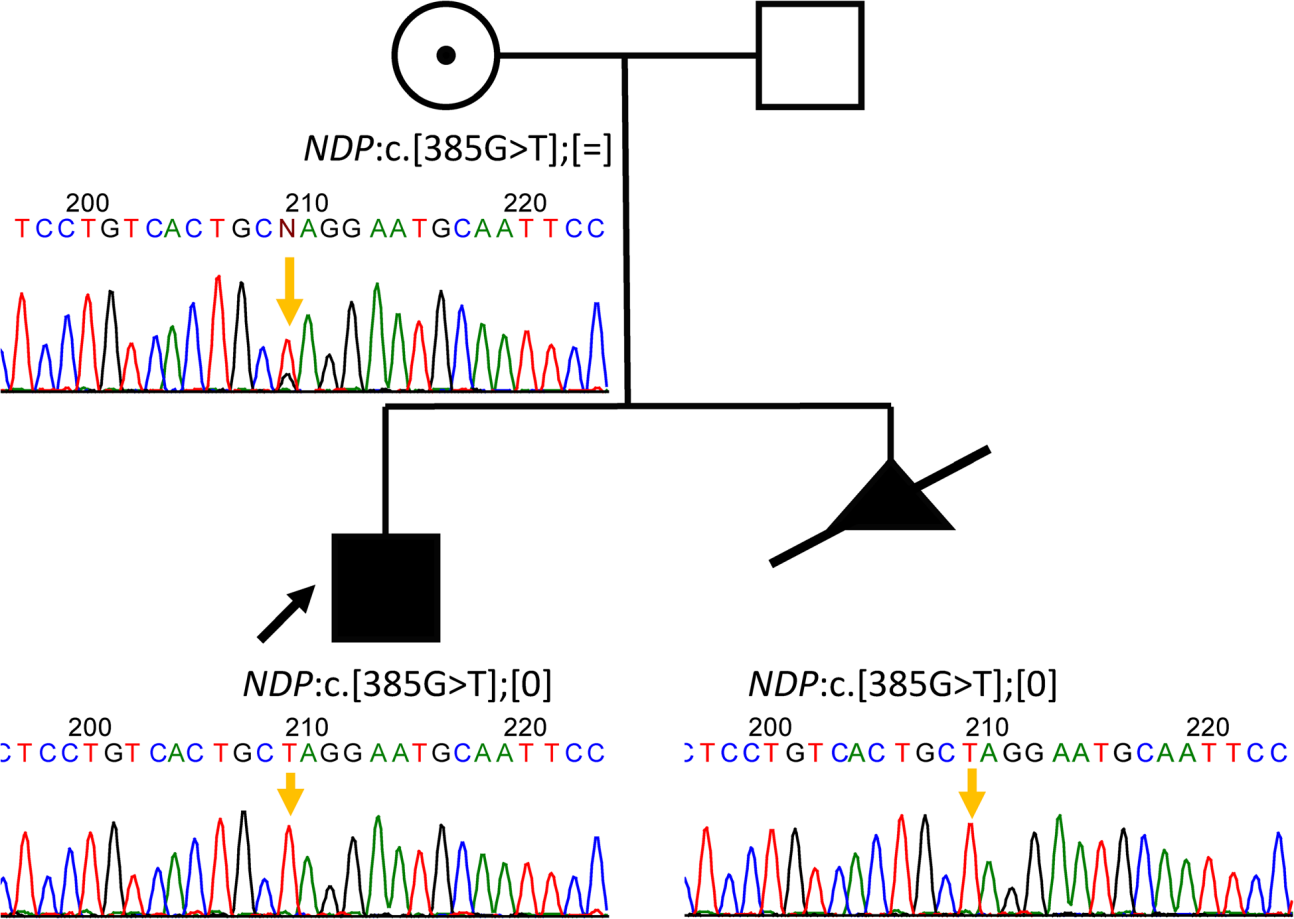
Pedigree of the family

A family referred to the Research Centre for Medical Genetics (RCMG) for risk-affected childbirth prognosis at 7–8 week of gestation because a previous child, a six-year-old boy, has congenital glaucoma, bilateral retinal detachment, and severe psychomotor delay and has had several ophthalmic surgeries (Fig. 1).

For the first time the proband (a full-term newborn) was examined by an ophthalmologist at the age of 4 months. Clinical presentation included roving eye movements and abnormal gaze behavior, but he had positive light perception and toy tracking. Intraocular pressure was 21 and 17 mmHg (OD and OS, respectively). B-scan ultrasonography revealed a total funnel retinal detachment, iris aplasia in both eyes, an anterior chamber of normal depth with an open angle, and undifferentiated trabecular meshwork, right eye lens opacity in all layers, bilateral vitreous fibrosis, and numerous fibrotic transocular folds behind the lenses. The proband was diagnosed as having the consequences of intrauterine uveitis, total retinal detachment, and secondary decompensated glaucoma. He later underwent several surgeries: 2 lensectomies, 4 vitrectomies, retinotomy, and subretinal aqueous drainage. No genetic test was performed at that time. The family never sought genetic counseling.

By the date when the family was referred for genetic counseling, the boy was blind in both eyes, had total bilateral retinal detachment, bilateral iris aplasia, left eye phthisis bulbi, pronounced delay in psycho-emotional development, lack of speech, and behavioral disorder.

Considering the ongoing pregnancy and the lack of a confirmed diagnosis, we performed NGS analysis of clinically relevant genes. Whole exome sequencing (WES) was performed using an BGISEQ-500 instrument with average on-target coverage 146× with MGIEasy Exome Capture V4 (BGI) for library preparation (Genomed Ltd., Moscow). Bioinformatic analysis was performed using an in-house software pipeline which included quality control of raw reads (FastQC tool v. 0.11.5) followed by read mapping to the hg19 human genome assembly (bwa mem v. 0.7.1), sorting of the alignments, marking duplicates (Picard Toolkit v. 2.18.14). Base recalibration and variant calling were performed with GATK3.8. Variant annotation was done using Annovar tool (v. 2018Apr16). Further filtering was performed by functional consequences and population frequencies according to the ACMG recommendations [1] as well as clinical relevance determined by Human Phenotype Ontology database [2]. The analysis lasted 42 days and revealed a novel hemizygous substitution, NM_000266.3(*NDP*):c.385G > T, p.(Glu129*), in the *NDP* gene, which was associated with Norrie disease (OMIM #310600) inherited in X-linked recessive mode. Subsequent validation of the affected child and his mother by Sanger sequencing confirmed the identified substitution. The substitution was not registered in control cohorts in the Genome Aggregation Database (gnomAD). The identified novel *NDP* sequence variant c.385G > T, p.(Glu129*), forms a premature termination codon (PTC). Although the variant affects only the most C-terminal portion of the protein (129 out of 133 amino-acid residues), it eliminates the ultimate cysteine-131 residue that is involved in formation of an intermolecular disulfide bond [3]. This should lead to the disruption of a very rigid structure of Norrin homodimer. Nearby PTC forming pathogenic variants have been described – p.Cys128* and p.Cys131* [4, 5]. It was previously shown that disruption of the dimer by either cysteine-to-alanine mutations of intermolecular disulfide bonds results in a loss of Norrin function [6, 7]. Norrin is an atypical Wnt ligand that can activate β-catenin signaling through its specific binding to the Frizzled4 (Fz4) receptor [8] to function as a ligand–receptor pair that controls vascular development in the retina and inner ear [6]. Pathogenic variants in the *NDP* gene are associated with a spectrum of conditions – from milder familial exudative vitreoretinopathy to the systemic Norrie disease [9].

The clinical exome sequencing in the proband motivated amniotic fluid testing in his pregnant mother at the 17th week of gestation, which revealed after additional 5 days that the fetus was male and hemizygous for the variant. The family decided to interrupt the pregnancy after the prenatal test at the 20th week of gestation. Complications in the form of profuse uterine bleeding developed during the pregnancy termination required hysterectomy due to medical necessity.

## Discussion and conclusions

Congenital vitreoretinopathy with retinal detachment is a highly heterogeneous group of disorders. Patients with genetically determined disorders such as familial exudative vitreoretinopathy, Norrie disease, or Coats disease, as well as non-hereditary retinopathy of prematurity or inflammation of the retina could have similar ocular manifestations [9]. Norrie disease is a rare X-linked recessive disorder of retinal blood vessel development. According to Orphanet database, ~ 400 cases have been described to date [10]. They are characterized by congenital vitreoretinopathy, avascularity of the peripheral retina and its secondary neovascularization, fibrosis, traction and retinal detachment, and blindness, which in about 25–50% of the cases, is accompanied by progressive mental retardation and/or sensorineural deafness [11, 12]. In addition, some patients have more complex phenotypes, including growth failure and seizure. Radiological examination of some patients with Norrie disease has demonstrated brain and cerebellar atrophy [13].

The cellular basis for Norrie disease is an insufficiency of Frizzled signaling, and more specifically the Frizzled-4 ligand Norrin. Norrin functions as an activator of the canonical Wnt/β-catenin signaling pathway in capillary endothelial cells in the retina and in other structures [12, 14]. Norrin is engaged in Müller glia in the retina, in stria vascularis and a capillary plexus of cochlea, in forebrain and midbrain astrocytes, and in cerebellum Bergman glia. Norrin is also required for blood–brain barrier integrity and maintenance [15]. Thus, Norrin can have developmental and homeostatic functions beyond the retina [16].

Nucleotide sequence variants in the *NDP* gene are associated with several congenital retinal pathologies with or without systemic involvement. To date, 167 mutations in the *NDP* gene have been registered in the Human Gene Mutation Database (HGMD) – among these 129 are associated with Norrie disease phenotype, 20 with familial exudative vitreoretinopathy, and 7 with retinopathy of prematurity. Most pathogenic variants of the *NDP* sequence (*n* = 100) are nonsense or missense mutations, and 80% are associated with Norrie disease [17].

In this study, we identified the pathogenic variant NM_000266.3(*NDP*):c.385G > T that introduces a PTC in amino-acid position 129 of Norrin. Molecular analysis allowed diagnosing Norrie disease in the patient. Severe ophthalmic phenotype with marked systemic involvement is believed to be linked to the absence of the ultimate cysteine, which forms the dimeric structure of mature Norrin and is in accordance with previous observations [18]. We suppose that in the case of earlier diagnosis, the family could have had much greater opportunity to give a healthy birth avoiding life-threatening complications, as well as averting the psychologic tragedy in the family.

Clinical polymorphism and variable expressivity of hereditary ophthalmic pathology severely complicates the establishment of an exact diagnosis and make diagnosis time- and cost-consuming. NGS appears to be the method-of-choice in complicated cases, which could substantially hasten the establishment of a diagnosis and genetic risk estimation. This is much more important in case of extremely rare diseases that are not well-known to physicians and ophthalmologists.

## Data Availability

The datasets used and/or analyzed during the current study are available from the corresponding author on reasonable request.

## Abbreviations

ACMG: American College of Medical Genetics and Genomics
HGMD: Human Gene Mutation Database
mmHg: millimetre of mercury
NGS: Next-generation sequencing
OD (oculus dextrus): The right eye
OMIM: Online Mendelian Inheritance in Man
OS (oculus sinister): The left eye
PTC: Premature termination codon
RCMG: Research Centre for Medical Genetics
WES: Whole exome sequencing
